# SARS-CoV-2: vaccinology and emerging therapeutics; challenges and future developments

**DOI:** 10.4155/tde-2021-0075

**Published:** 2022-02-23

**Authors:** Adedapo Adesokan, Mohammad A. Obeid, Aisha F Lawal

**Affiliations:** ^1^PreciseMed, Glasgow, UK; ^2^Department of Pharmaceutics & Pharmaceutical Technology, Faculty of Pharmacy, Yarmouk University, Irbid, Jordan; ^3^Consultant Paediatrician with special interest in Vaccinology & Infectious Diseases, Children’s Specialist Hospital, Ilorin, Kwara State, Nigeria

**Keywords:** challenges, COVID-19, future developments, mutated strains, nanoparticles, nanotechnology, vaccines

## Abstract

As SARS-CoV-2 emerge, variants such as Omicron (B.1.1.529), Delta (B.1.617.2), and those from the United Kingdom (B.1.1.7), South Africa (B.1.351), Brazil (P.1) and India (B.1.6.17 lineage) have raised concerns of the reduced neutralising ability of antibodies and increased ability to evade the current six approved COVID-19 vaccine candidates. This viewpoint advocates for countries to conduct prior efficacy studies before they embark on mass vaccination and addresses the role of nanoparticles as carrier vehicles for these vaccines with a view to explore the present challenges and forge a path for a stronger and more viable future for the development of vaccines for SARS-CoV-2 and future pandemics. We also look at the emerging prophylactics and therapeutics in the light of ongoing cases of severe and critical COVID-19.

## Introduction

The coronavirus is a member of the virus family reputable for causing the common cold, severe respiratory illnesses and death. Pathogenic examples include Middle East respiratory syndrome (MERS)-related coronavirus, severe acute respiratory syndrome coronavirus (SARS-CoV), severe acute respiratory syndrome coronavirus 2 (SARS-CoV-2) and so on. Animals, including camels, cattle and bats, among others, are known to be reservoirs [1]. Recently attributable to coronaviruses is SARS and MERS, which are two major outbreaks in the Far East [2,3]. The advent of COVID-19 began in Wuhan, China, on 30 December 2019 [4], before it rapidly degenerated to a global disease with immense significance on global travels and shut down local endeavours throughout the world. There are four major routes of transmission attributed to SARS-CoV-2: direct spread through the air, physical contact with a carrier, indirect contact with a SARS-CoV-2 contaminated item, and possibly from droplet and airborne transmissions that are breathed in from coughs, sneezes and aerosolization, when the atomised virus becomes suspended in airflow [5].

Human nasal linings comprise of a large number of cell receptors called ACE2. The presence of ACE makes the nasal epithelium a good portal of entry for SARS-CoV-2 [6]. The SARS-CoV-2 structure has four major proteins, namely spike (S) glycoprotein, nucleocapsid (N) protein, membrane (M) glycoprotein, envelope (E) glycoprotein and other accessory proteins [7].

The S protein’s key role may be found in the viral attachment, fusion, entry and transmission of SARS-CoV-2. It functions by attaching itself to the ACE2 receptor to provide a means of entry for the SARSCoV-2 virus to enter the human body [8]. The main immunogenic target of the currently approved COVID-19 vaccines is the S protein [9].

The endoplasmic reticulum–Golgi region houses the N protein. It binds to RNA to perform the critical role in the viral genome associated with the viral replication cycle. It is also involved in the cellular response of the host cells to viral infections [7,10].

M protein plays an important role in the determination of the shape of the virus envelope. The E protein plays a critical role in the production and maturation of the virus. It is the smallest of all major SARS-CoV-2 structural proteins [7].

At the moment, the most topical issue on COVID-19 globally is the new mutant strains that are capable of evading the immune system and somewhat resistant to the current vaccines [11,12]. As viruses reproduce, ‘copying errors’, otherwise known as genetic mutations, emerge to cause changes that affect the surface, shape and/or proteins of the virus [13]. In as much as the S protein is essential for the receptor binding and membrane fusion, it is also targeted by the neutralizing antibodies. The E protein is responsible for virus infectivity, whereas the M protein, being the most abundant protein found in SARS-CoV-2, forms the viral envelope that is constantly interacting with the E protein [14].

**Figure 1.**
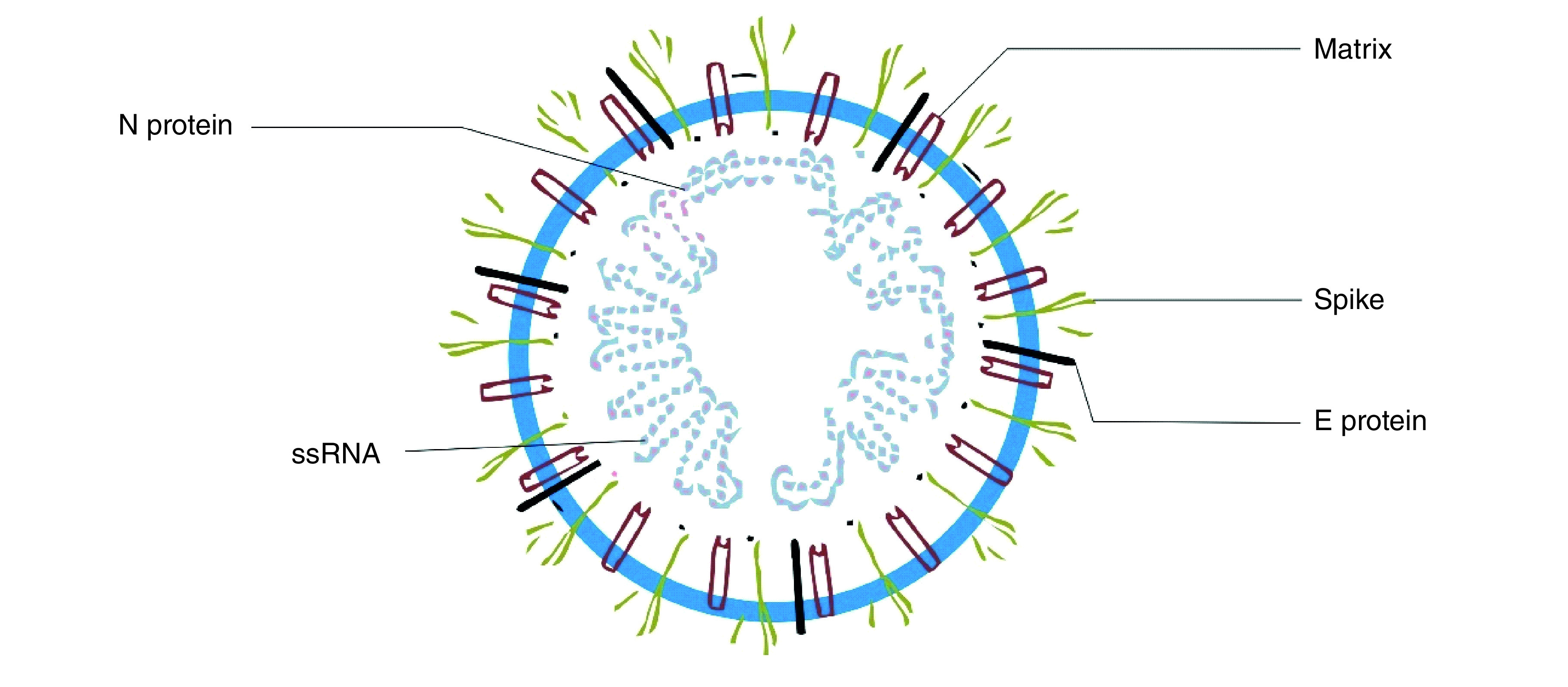
SARS-CoV-2 structure.

Figure 1 presents the structure of SARS-CoV-2.

**Figure 2.**
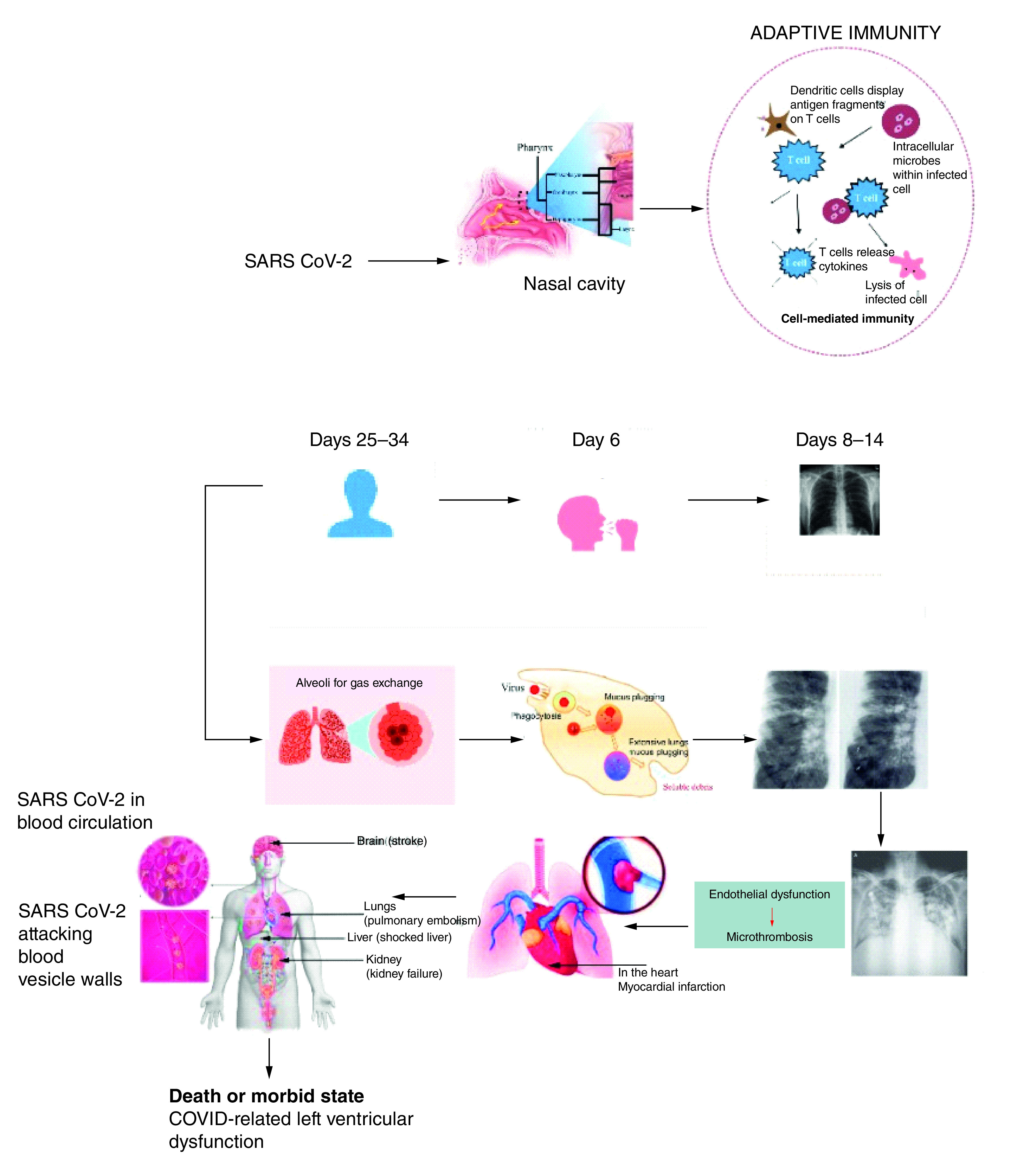
SARS-CoV-2 disease progression.

The process of rapid cell replication and reprogramming is immediate soon after SAR-CoV-2 infects a host after gaining an entry through the nasal epithelium cells of a human host [15,16]. Within this host, SARS-CoV-2 would target the ciliated and goblet cells where subsequent viral shedding and multiplications facilitate an extensive volume of viral load buildup within the upper respiratory tract. In terms of disease progression, as illustrated in Figure 2, in severe-to-critical COVID-19 patients, the lungs are filled with replicating viruses and neutrophils attack, which are capable of killing the virus-infected lung cells. At this point, the patients will develop a productive cough containing mucus-laden, dead, virus-infected lung cells, neutrophils and the protein-rich fluid [17].

Also accompanying these symptoms are dyspnoea and bilateral diffuse pneumonia of both lungs resulting from direct damages to the lung parenchyma linings induced by the immune cells. These damages are predicated on the effect of cell hyperinflammation resulting from the overreaction of cytokines because of the replicating SARS-CoV-2. This process is referred to as the cytokine storm, which is indicative of severe-to-critical COVID-19 infection [18]. The cytokine storm precedes acute respiratory distress syndrome, microthrombosis and subsequent multiorgan failures that follow.

## Current vaccine technologies

**Figure 3.**
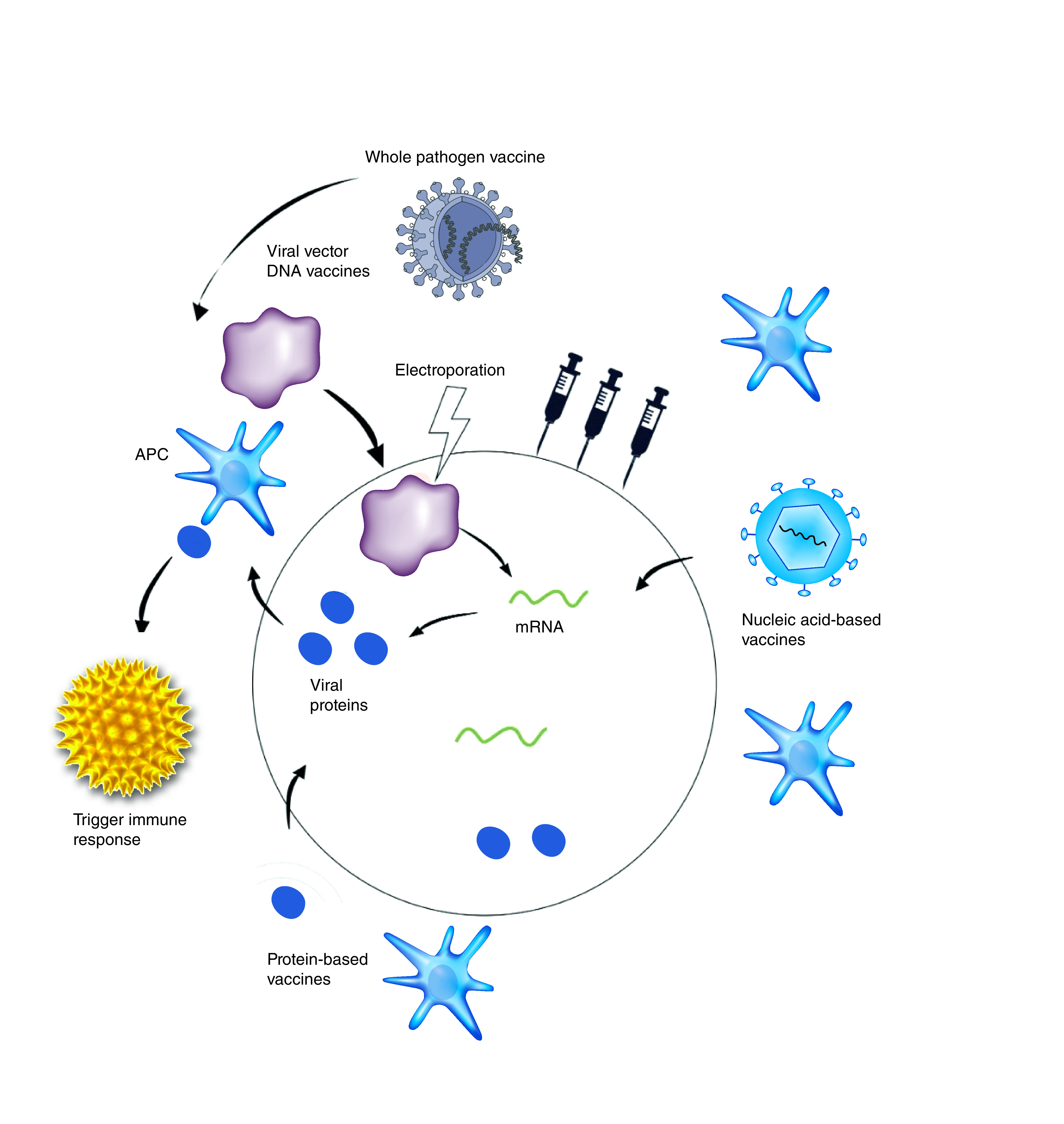
SARS-CoV-2 vaccine approaches.

The scientific development and modular creation of COVID-19 vaccines have taken different pathways and approaches. Most prominent are use of the genetic material (nucleic acid vaccines), the viral vector, the whole pathogen (whole-microbe approach) and the protein approach. Figure 3 describes some of the approaches.

### Whole-microbe approach

Otherwise known as the whole-pathogen vaccine, the whole-microbe approach comprises the development of an inactivated vaccine, a live attenuated vaccine or, in a modified version, a viral vector vaccine [19]. The whole-pathogen vaccine development approach is the oldest and best-known method of vaccination because it deploys the weakened or inactivated entire disease-causing pathogen to elicit an immune response following vaccine administration.

The inactivated vaccine approach involves inactivating a whole SARS-CoV-2 virus using heat, radiation or chemicals [20]. This approach was previously successful in the development of flu and polio vaccines with good therapeutic responses against the two diseases after vaccination. The development of the Chinese Sinovac and Sinopharm vaccines, as well as the Scottish SARS-CoV-2 candidate vaccine Valneva, among others, have their origins in the inactivated vaccine approach.

The live-attenuated vaccine derives its basis from a weakened-virus approach. Examples of live-attenuated vaccines currently used in the UK schedule include the rotavirus vaccine, MMR vaccine, nasal flu vaccine, shingles vaccine, chickenpox vaccine and BCG vaccine [20]. The major drawback to this approach is the contraindication among people with profound immunosuppression.

### Viral vector vaccine approach

The viral vector vaccine approach involves the use of a genetically modified, safe virus that has lost its ability to replicate to deliver certain genetically coded instructions to generate specific immunogenic proteins. A typical example is the AstraZeneca Oxford COVID-19 vaccine that used the ChAdOx1 vaccine technology using adenovirus type 5 that was safely isolated from chimpanzees. The incorporation of the genetic material into the viral vector led to the development of the vaccine (AZD1222) currently in use against SARS-CoV-2 [21]. The virus was modified genetically into DNA, and later it was used to form the mRNA that is specific to SARS-CoV-2 spike proteins that can penetrate the ribosome of the human cytoplasm. The newly formed protein product is capable of triggering an antigen presentation, which ripples off to forge a T cell memory response and the production of humoral antibodies by B cells. The ChAdOx1 vaccine technology is the progenitor of candidate vaccines against the flu, Zika and MERS pathogens.

### The genetic approach (nucleic acid vaccine)

The nucleic acid vaccine approach involves the use of genetic material that encodes instructions for making specific immunogenic proteins [22]. These vaccines delivers a specific set of instructions in the forms of nucleic acids such as DNA, mRNA, or siRNA to specific human cells which will be then translated inside the cells into specific protein to trigger memory T-cell responses and the production of humoral antibodies. The Pfizer-BioNTech and Moderna COVID-19 vaccines emerged from this approach.

### Protein-based approach

The protein-based COVID-19 vaccines are recombinant versions of the whole spike protein, fragments of the spike protein, or many protein molecules packed into nanoparticles (NPs). These vaccines also feature an adjunct compound that primes frontline immune cells to trigger efficient immune responses when exposed to the protein antigen. Protein-based vaccines are slower to develop compared with nucleic acid-based, viral vector or whole-pathogen vaccines, but they tend to be safe and effective. Examples include the Novavax and Sanofi/GSK COVID-19 candidate vaccines.

### Safety issues & the biodistribution of vaccine delivery

The biodistribution and safety of the COVID mRNA Pfizer vaccines are linked to concerns about whether the commercial manufacturing standards are being compromised. In an email leaked to BMJ dated 23 November 2020, the issue of integrity and doubts about adherence to vaccines specifications were under scrutiny. Concerns were raised there is* “a significant difference in % RNA integrity”* between the clinical batches and proposed commercial batches – from around 78% to 55% [23]. EMA has issued a response authorised on the Pfizer-BioNTech’s vaccine, stating “*the quality of this medicinal product, submitted in the emergency context of the current (COVID-19) pandemic, is considered to be sufficiently consistent and acceptable”* [24].

Issues revolving around quality, integrity and compliance show the complexities of novel mRNA vaccines, more so that specific regulatory guidance for mRNA-based vaccines are yet to be established.

Areas of regulation should include the quantification and integrity of mRNA and those of the carrier lipids. Other parameters needed would be the accuracy of the specific charge and distribution of particle sizes. For a charge to be valid, it must be specific because a wrong charge could incite mRNA degradation. Furthermore, the right encapsulation is necessary prior to uptake into cells. This also helps to prevent endosomal escape in the mRNA for the attainment of the transfection within the host cells after intramuscular vaccine administration. All these parameters are commonly evaluated at the preclinical drug development phases.

#### RNA instability

RNA instability is one of the most crucial variables in mRNA vaccines. The challenge posed by RNA instability is one of the biggest hurdles that nucleic acid therapeutics researchers face in their efforts to develop nucleic acid-based vaccines. RNA instability necessitates the need for a stringent cold chain requirements and the right encapsulation of the mRNA in lipid nanoparticles (LNPs). The ultimate success of the process is measured by the encapsulation efficiency ratio achieved during the development phase. Post uptake into cells, a whole, unbroken mRNA molecule is essential for the potency of any nucleic acid-based vaccine. Any minor degradation reaction along the line by RNAses or along an mRNA strand can significantly impair or retard the correct translation or functionality of the affected strand, which can result in deficient expression of the target antigen [24,25].

#### Pharmacodynamics and pharmacokinetics

Primary pharmacodynamics studies *in vitro* done to confirm the efficacy of the BNT162b2 (V9) RNA-based product in terms of protein expression, transfection frequency from BNT162b2 and cell surface expression of the SARS-CoV-2 P2 S protein antigen were assessed in the preclinical studies carried out in the course of development of the BNT162b2 vaccine. SARS-CoV-2 P2 S protein adequate expression on the cell surface was also confirmed following BNT162b2 (V9) transfection of HEK293T cells [24].

#### In vivo pharmacodynamics study

In an *in vivo* study after the intramuscular administration of BNT162b2 (V9) in mice and nonhuman primates, humoral and cellular immune responses were studied. Nonhuman primates were used in the study, because as a higher-ordered species, they are most related to humans in terms of immune responses. Antigen-specific immune response following BNT162b2 vaccination in Wistar Han (WH) rats were also shown in the course of the studies. Cytokine profiling using Multiplex analysis revealed high levels of the Th1 cytokines IFN-γ and IL-2, but minute amounts of the Th2 cytokines IL-4, IL-5 and IL-13 were detected after re-stimulation [24]. The study also revealed an elevated secretion of TNF-α, GM-CSF and IL-1β; IL-12p70 and IL-18 were recorded after re-stimulation [24]. This study showed the S-specific IFN-γ levels indicating significant T-cell responses. It also showed a high frequency of CD4^+^ T cells that produced IFN-γ, IL-2 or TNF-α, signifying a favourable Th1-biased response materialised after the BNT162b2 (V9) immunization [24].

#### Pharmacokinetics

In pharmacokinetics studies, the two novel LNP excipients in the Pfizer mRNA COVID vaccine ALC-0315 (aminolipid) and ALC-0159 (PEG-lipid) in plasma and liver were investigated. The study also examined their elimination and metabolism in rats, as well as the biodistribution of a LNP-formulated surrogate luciferase RNA in mice intravenously and the biodistribution of a [3H]-labelled LNP-mRNA formulation in rats intramuscularly [24].

Different time points *in vivo* luciferase expression at the injection sites and in the liver region were also ascertained, indicating drainage to the liver. The luciferase expression was short-lived, and it reduced over time. The signal however decreased slowly during the first 72 h [24]. This was consistent with what is expected in an efficient mRNA vaccine.

#### Radioactivity

Detection of radioactivity signal in most tissues from the first time point (0.25 h) was revealed, indicating that the two major sites of distribution are the injection site and the liver. Most tissues revealed low levels of radioactivity detection, with the greatest levels of radioactivity seen in the plasma at 1–4 h post-dose. Over 48 h, the distribution was mainly observed to the liver, adrenal glands, the spleen and the ovaries, with maximum concentrations observed at 8–48 h post-dose [24].

## Nanoparticles & novel COVID-19 mRNA vaccine development

The therapeutic application of mRNA-based vaccines is being explored as an effective vaccine development formula against cancer and infectious diseases. The mRNA-based vaccines are considered safer, more cost effective and much easier to develop than live vaccines [26,27]. The combination of low cost and race to overcome the pandemic makes mRNA vaccines a better option when compared with the traditional vaccines. Despite these advantages, mRNA vaccines have some drawbacks, and the biggest problem is how the mRNA molecules are prone to degradation by nucleases and thus intrinsically are unstable after administration. In addition, the mRNA molecules are negatively charged molecules with large molecular weight that limits their uptake by the cells and reduces their availability in the cytoplasm of the target cells where they are expected to exert their effects [28]. Many preclinical studies tailored along these lines heralded the emergence of the approved mRNA SARS-CoV-2 vaccines. The goal of each study was to enable the scientists to identify the most suitable carriers that can carry the vaccine to the host cell efficiently [29]. This article seeks to provide information on how this challenge of vaccine delivery can be overcome using nanotechnology.

### Nanotechnology

NPs are capable of acting as efficient delivery systems for mRNA-based vaccines. A host of NPs have been studied as agents of antigen delivery and also for their ability to offer protection against myriad health conditions. Upon administration, the NPs can transport, protect and deposit the loaded mRNA molecules into the target cells [30]. In today’s new delivery methods for vaccine development, nanotechnology stands out as unique in its abilities to guide vaccine contents into targeted cells in a human body efficiently [31]. Reported examples of NPs in this field include bacterial toxic liposome for cholera vaccination, viral protein gold NPs for vaccination against mouth and foot disease, and viral protein polypeptide NPs for coronaviruses [32–34].

**Figure 4.**
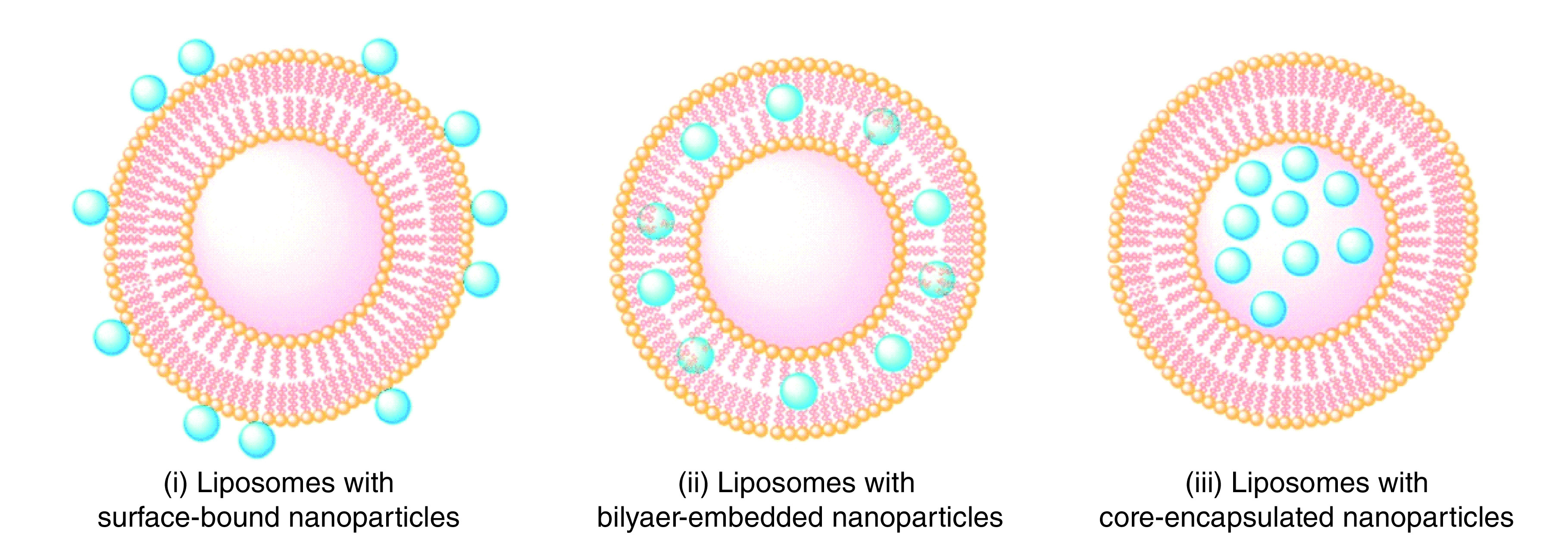
Different configurations of liposomes in nanoparticles.

Gold, carbon and silica are the most commonly used types of NP; they are inorganic NPs. Advantageous features of NPs include low production cost, reproducibility and safety [35]. Polymeric NPs are a separate type of NP, and they have gained more attention recently because of their ease of preparation, biodegradability, biocompatibility and reduced cytotoxicity [36]. However, among the different types of NPs, lipid-based NPs such as liposomes and niosomes are among the most investigated types of NPs in the field of vaccine delivery. These NPs consist of an aqueous core surrounded by one or more lipid bilayer formations [26], as shown in Figure 4.

Liposomes are composed of phospholipids with a bilayer structure, and they are deployed in the delivery of many active drugs and antigens. NP technology as an adjunct can enhance immunity by increasing the absorption of an antigen. The NPs are also capable of holding multiple epitopes of the antigen, which creates the endocytosis of the antigens by macrophages [37]. When phospholipids in liposomes are replaced with nonionic surfactants, the emerging products are liposome-like NPs, known as niosomes. In addition, the niosomes consist of a bilayer structure that is surrounded by an aqueous compartment – a feature that makes niosomes more chemically and biologically stable than liposomes [38].

When NPs are used for vaccine delivery, they serve as adjuvants and act as carriers to prevent antigen degradation in the body; they also improve antigen stability. Furthermore, NPs have the advantage of being able to encapsulate more than one agent at a time. The co-encapsulation of the antigen with an immune-stimulatory agent (adjuvant) within the same NP will improve the effect of the vaccine. After administration, phagocytosis of these NP-based vaccines by the antigen-producing cells will result in an increase of MHC-1 levels, which will eventually promote cytotoxic T-cell recruitment [39]. In addition, control of NP size, shape and charge can control antigen circulation in the body, biodistribution, bioavailability and specificity (by targeting specific biological barriers).

Furthermore, among the different vaccines that are being developed, two interesting types of mRNA-based vaccines gained emergency approval and are in clinical use. The first one was developed by Pfizer and BioNTech, and is referred to as BNT162b2. This vaccine contains lipid NPs loaded with mRNA molecules that encode for the spike proteins, which are presented on the outer surface of SARS-CoV-2. The administration of this vaccine culminates in the production of antibodies against the viral S protein [40].

Mitigating against the progress of mass vaccination and vaccine uptake worldwide response is the fact that SARS-CoV-2 can mutate at an approximate rate of one to two mutations every 30 days [41]. The first mutant variant (D614G mutation) was identified in March 2020 and then a team of virologists led by Korber, Montefiori reported that the D614G mutation transmission was exponentially increasing in the western world [42,43]. The D614G rapidly emerged as the dominant SARS-CoV-2 lineage in Europe and gained rapid dominance in the USA, Canada and Australia [44]. In the UK, the B.1.1.7 variant, discovered in the fall of 2020, was the first mutant strain of specific concern. The B.1.1.7 variant posed the risk of increased transmissibility and was said to be 56% more contagious than the original strain of SARS-CoV-2 [45].

**Figure 5.**
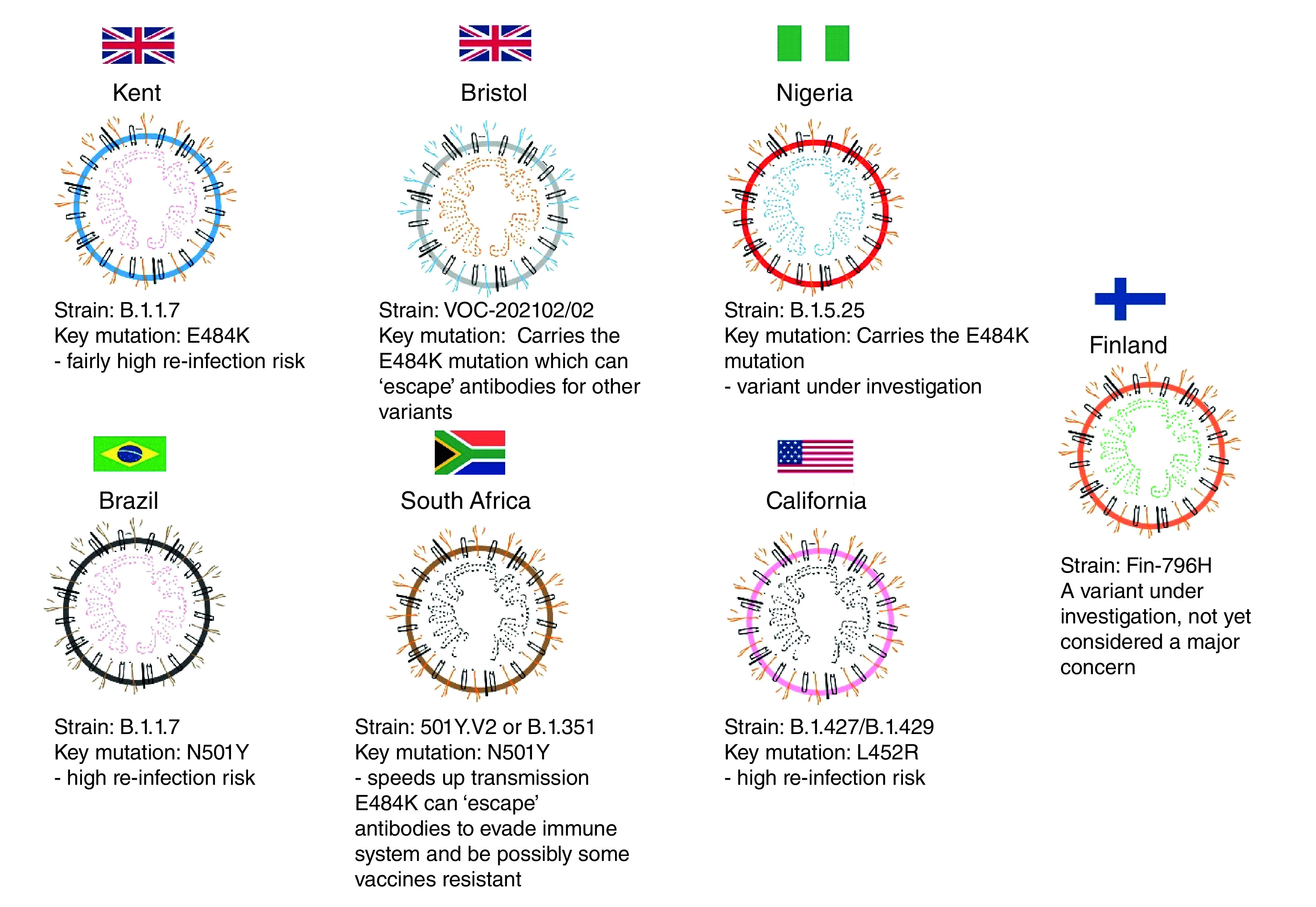
SARS-CoV-2 mutations and corresponding strains of concern.

In terms of the efficacy of current vaccines, Moderna and Pfizer products have proven effectiveness of up to 95% against B.1.1.7. The Novavax vaccine is about 86% effective against B.1.1.7 [46,47]. Other emerging mutant variants of concern are the Bristol variant E484K, which enables the virus to evade vaccines to an extent. The process resembles an ‘escape mutation’ because it enables the virus to slip past the body’s immune defences and can substantially increase the amount of the serum antibody needed to prevent infection in the cells [46]. The South African strain B.1.351 is a variant of E484K. Conducted clinical trials in South Africa by Novavax and Johnson & Johnson discussed the comparable effectiveness of their new vaccines against the high prevalence of E484K mutant virus. Novavax reported a 60% efficacy compared with 86% in the UK [36,48]. The Nigerian variant’s genome is very similar to the Kent variant, B.1.1.7, and its spike protein carries the E484K mutation, similar to the South African and Brazilian variants. As of the writing of this article, it remains under investigation and was yet to show serious clinically significant concerns. The Brazilian variant is also noted to carry the S protein mutation E484K and was first detected in Rio de Janeiro in October 2020 [49]. Recently, most cases of COVID-19-related hospitalization globally were due to the Delta variant of the mutation known as B.1.617.2; it was first identified in India in December 2020. A recent Chinese study revealed that viral loads from the Delta strain have proved to be 1000-times more infectious than other variants [50]. A summary of global trends of SARS-CoV-2 mutants is shown in Figure 5.

Adenovirus or adeno-associated virus (AAV) vectors were used to develop the AstraZeneca and Janssen Ad26.CoV2. S COVID vaccines and that's why they are refered to as adenovirus vector-based vaccines. The adenovirus vaccine carriers are immunogenic and highly efficient vaccine vectors. In comparison to adenovirus vectors, the AAV vectors are poorly immunogenic and are therefore more useful in gene replacement therapy rather than as vaccine carriers. The viral vector vaccines have their immunogenicity reduced by the presence and actions of pre-existing neutralizing antibodies [51].

AAVCOVID is a spike-gene-based COVID-19 vaccine candidate; it is a single AAVCOVID dosed vaccine that in monkeys has shown protection from SARS-CoV-2 with neutralizing antibody levels maintained at peak for a year. They are scalable and remain potent even if not refrigerated or kept at ambient temperature for 30 days [52].

These replication-defective adenovirus vectors have consistently induced potent transgene product-specific B- and T-cell responses. The vectors have been used for developing the current viral vector COVID vaccines. The immunogenicity of adeno-associated based virus vectors was poor before research was done on a hybrid vector from rhesus macaques and chimpanzees. The virus vectors have proven their ability to induce potent innate immune responses, with a good example being the AstraZeneca vaccine that comes from chimpanzee replication-defective adenovirus vectors.

In terms of lipid carriers and immune interactions, the Pfizer mRNA COVID vaccine, for example, has its LNP carrier ([4-hydroxybutyl] azanediyl) bis [hexane-6, 1-diyl] bis [2-hexyldecanoate]), 2 [(polyethylene glycol)-2000]-N, N-ditetradecylacetamide, 1,2-distearoyl-sn-glycero-3-phosphocholine, and cholesterol) encapsulated to protect the mRNA from degradation. In terms of a specific immune reaction to the NPs, LNPs promote protein translation in lymph nodes and ensure target delivery to lymphatics. Dendritic cells engulf LNP in lymph nodes, which subsequently produce and present the antigen to T cells for activation of the adaptive immune response [53,54]. This results in the production of significant levels of S protein, and they usually trigger innate sensors through the intrinsic adjuvant activity of the vaccines. The synergistic effects result in the production of type I interferon and the release of multiple pro-inflammatory cytokines and chemokines.

Table 1 shows mutated SARS-CoV-2 strains of concern, their characteristics and their susceptibility to vaccines in a global context.

**Table 1.** Mutated SARS-CoV-2 strains of concern, their characteristics and their susceptibility to vaccines.

	Kent	Indian	Bristol	South African	Nigerian	Brazilian	California	Ref.
Strain number	B.1.1.7	B.1.6.17 (double mutation)	E484K	B.1.351/501Y.V2	B1.5.25	B.1.1.28	B.1.427/B.1.429: L452R mutation	[55]
Vaccine effect	Vaccines effective	Evades immune system	Evades vaccines easily	Evades vaccines easily	Evades vaccines easily	Evades vaccines easily	Considered a major concern with disease burden; evades vaccines	
Morbidity burden	Moderate increased risks of reinfection	Increased transmissibility	Increased risks of reinfection	Increased risks of reinfection	Variant under investigation	Increased risks of reinfection	Increased risks of reinfection	

A South African AstraZeneca Oxford vaccine study with 2000 young and healthy respondents [56] reported that the vaccine failed to protect against mild and moderate SARS-CoV-2 disease caused by the new 501Y.V2 variant. The 501Y.V2 variant accounted for about 90% of cases in South African and was also found to be predominant among HIV-negative people. This finding is not particularly concerning, as the study was carried out on a small group of patients and did not take into consideration vaccine efficacy against severe infection by COVID-19, ICU hospital admissions and deaths. However, of concern is Chile’s vaccination story. The WHO dashboard revealed that there have been 1,837,390 confirmed cases of COVID-19 reported to the WHO, with 39,289 deaths from 3 January to 11 January 2021. Assuming every person needs two doses, that is enough to have vaccinated about 119% of the country’s population, with booster doses accounting for the above 100% percentile value. Largely, Chileans are being vaccinated using the Chinese Sinovac vaccine with efficacy documented to be 50.4%, according to latest reports from Brazil [57]. As of 11 January 2022, no phase III trial data for any of the Chinese SinoVac vaccines have been officially published in a peer-reviewed journal. It is worth noting that in December 2020, Turkish health officials reported through interim trial data that Sinovac’s CoronaVac was 91.25% efficacious at preventing symptomatic COVID-19 among a trial group of 1322 respondents [58].

Indonesia-based health officials reported that the Sinovac vaccine being trialled in their country was 65% effective [59]. In India’s case, the world watched in horror as COVID-19 wreaked havoc in the winter and into spring of 2021, as the world’s largest vaccine-manufacturing nation became the COVID epicentre of the world yet had vaccinated only 1% of its population as of the spring of 2021. When the nation was worst hit by COVID-19 during the first half of 2021, India reported more than 208,330 deaths from 3 January 2021 to 27 April 2021 [60], with more than 300,000 new cases being reported daily.

The most recent lethal global wave of COVID-19 disease was attributed to the Delta variant. The Delta variant, which is otherwise known as B.1.617.2, contains multiple mutations with unbanning ability to evade the immune system. Although the current vaccines provide protection against the Delta strain and with mixed reports thus far on Omicron, the current global strain, the vaccines appear to be slightly less effective against the latter, and this accounts for breakthrough infections being witnessed globally despite patients being fully vaccinated. Thus, the South African and Chilean scenarios raise the question of whether pre-mass vaccination efficacy matching studies should be carried out on candidate vaccines to determine predominant variants in respective countries around the world before mass vaccination, to ensure optimal efficacy.

In the face of these worrisome mutations, experts are stating that the vaccines would need to be redesigned and tweaked to offer better protection against new variants in the coming months [61]. No revamped or redesigned COVID-19 vaccine is in clinical use yet, but vaccine development researchers are working on the possibility of substituting the old versions of the spike protein that had been created based on the earlier virus identified in Wuhan, China. The goal of the substitution is to modify the molecules that have more recently been identified in new variants. However, COVID-19 vaccine development researchers should determine if such changes would instigate a ‘knock-on’ effect capable of altering immune system response to the new vaccine. The knock-out effects would necessitate a need for new clinical trials with attendant costs. In the unlikely event that vaccine revamping is costly, scientists also may potentially include both the new variants of concern and the old forms of the spike protein to create a single, multivalent vaccine.

Moderna is mooting the idea to update their mRNA vaccine; this is geared at matching the SARS-CoV-2 virus spike mutations seen in the 501Y.V2 variant through a third dose of their original vaccine. Moderna, a well-known reputable biotech company, not only is seeking to test the effectiveness of the third dose but also is considering the possibility of bringing to the market a multivalent vaccine. Related research studies are currently ongoing using the novel multivalent NP-based vaccines on a hamster animal model, with a view to ascertain their efficacy against SARS-CoV-2 after a single vaccination dose [61]. Currently, a third booster vaccine dose has been initiated for the immunocompromised and frontline health workers in Israel, Hungary, the USA, UK and many other western nations before the rest of the population. It is worthy of note that seasonal influenza vaccines are redesigned periodically, as the strains are monitored regularly for emergence of new strains that limit vaccine efficacy. This is done by revamping the updated vaccines to reach acceptable levels of neutralizing antibody responses, to match desired efficacy, despite emergence of new strains of influenza viruses.

In terms of therapeutics to treat and prevent SARS-VoV-2 infections, dexamethasone from the RECOVERY trial has proved to be pivotal in clinical use in the UK. It is recommended in the UK in hypoxic patients with PCR-positive COVID; it limits the hyperinflammatory process and aids recovery. There is no evidence to suggest any benefit when used in nonhypoxic SARS-CoV-2 severe patients. The other steroid being investigated in early-stage disease is budesonide, although this is yet to adapt to the national treatment guidelines in the UK. In the USA, several monoclonal antibodies are being trialled in the intensive care unit to improve survival in patients on ventilators with critical SARS-CoV-2 infections.

In terms of prophylaxis therapeutics, Merck has developed molnupiravir, with the UK being the first country to approve its use in preventing severe disease. Pfizer’s Paxlovid is said to prevent hospitalisation with severe COVID infection by 90% and has been authorised by the Medicines and Healthcare products Regulatory Agency (MHRA) in the UK for adults who could be vulnerable to coronavirus due to age, weight or a prior chronic illness.

This article is canvassing for more innovative solutions in the treatment of severe to critical COVID infection, as well as development of medical devices as prophylaxis as the world continues to witness few numbers of intensive care unit admission of triple-vaccinated individuals. These prophylactic innovative solutions could be in the form of antiviral facemasks, barrier gels, nasal patches and nasal sprays, among others.

### Emerging COVID vaccine development approaches

One way to tackle the limiting impact of mutations on vaccine effectiveness globally is to review the approach to vaccine development. Whole-microbe vaccines are traditional, conventional vaccines, made from live viruses that have been weakened or inactivated. In particular, the weakened vaccines have historically generated very strong immune protection. In light of persistent SARS-CoV-2 mutations of great concern worldwide, mucosal vaccines and whole-microbe vaccines might be worth investigating more closely for efficacy against a wide variety of mutated viruses in future pandemics. Whole-microbe vaccines are well known for ease in manufacturing using well established methods, cheap cost of manufacturing, ability to provide strong immune responses, and eliciting of both T-cell-mediated and B-cell-mediated immune responses. The task of combating these mutations requires precision, because the whole-pathogen vaccines may be more advantageous since they present many viral antigens simultaneously. When the latter takes place, if a part of the virus such as the S protein presents as an antigen, a mutation that is capable of changing the shape and structure could take place. Where present, other antigens such as M, E or N proteins, accessory proteins and other immunogenic SARS-CoV-2 antigens would theoretically ensure that the vaccine remains effective in their virus-neutralizing action, despite the mutations. In addition, by providing a wider range of antigens, in theory, these whole-pathogen vaccines would afford better cross-protection against virus variants, should mutations of concern persist globally. Another advantage of whole-microbe vaccines is the fact that they might work faster, as they do not require ribosome processing to build up the antigen, so immunity is conferred quite early after vaccination, implying that the prime-boost interval and optimal interval is shorter than it is with mRNA, viral vector or protein-based vaccines. However, on the downside, these types of vaccines, especially the live-attenuated ones, have the potential to trigger new diseases in rare cases and are unsuitable for severely immunocompromised individuals, as they are only weakened in development and retain the potential to grow, replicate and even cause harm.

A futuristic approach would be to develop nasal mucosal vaccines, which would be unique in the sense that they would generate mucosal immune responses such that they stop the virus from replicating right at the point of entry, prevent spread and protect against severe disease even before mutated viruses have the opportunity to evade the immune system and vaccines. Historically, childhood infections have been largely attributed to respiratory-borne viral pathogens which gain entry through human respiratory mucosal surfaces to cause infections. The parenterally delivered vaccines, however, are not magic bullets capable of inducing protective immunity at these mucosal surfaces. The mismatch is largely due to the fact that there are differences in the mucosal surfaces, variabilities in the mucosal ciliary clearance and other challenging factors that naturally exist in humans [55].

Due to advancement in nanotechnology, one way to circumvent this limitation would be to develop the mucosal vaccines in NPs while keeping in mind the complex human respiratory epithelium mucosal anatomy. These proposed mucosal vaccines would be carried in niosomes or liposomes to aid penetration and increase retention time using permeation enhancers [62]. Should this theory become a reality and produce a viable lead vaccine candidate, it would be highly advantageous because mucosal vaccines allow antigens to interact with the mucosa-associated lymphoid tissue to generate mucosal and systemic immunities simultaneously. The induced mucosal immunity is capable of preventing and inhibiting the viral pathogen on the mucosal surface before it can encourage or foster any type of disease progression. With a good formulation strategy, these proposed mucosal vaccines may reinforce the barrier activity defence at mucosal surfaces in addition to proffering immunity. The mucosal administration of vaccines offers a protective layer and shields against the fear and pain of a needle, which is common in children and also in some adults. In addition to overcoming needle phobia, mucosal vaccines also eliminate injection site pain, local redness and inflammation, all of which are examples of common discomforts associated with the administration of COVID-19 vaccines. Based on the foregoing, mucosal vaccination is highly recommended for immediate production and distribution after scientists tackle the challenges identified with vaccine delivery across mucosal surfaces, the potential knock-on effects of novel vaccines and how to alter the immune system response that would accompany mucosal vaccinations [55].

### Neutralizing antibody escape mechanisms in COVID-19 mutant strains

In a pandemic, most mutations are of nonsevere clinical significance and are found in the SARS-CoV-2 genome as mildly deleterious, swiftly purged or relatively neutrally harmless, but occasionally a small proportion will effect a significant disease course with increased infectivity by affecting functional properties in the genome. The high-effect mutations contributes to virus adaption and a fitness can occur in very few cases when compared with the more ubiquitous tolerated low-effect or no-effect ‘neutral’ amino acid changes [63]. Since the emergence of SARS-CoV-2 in December 2019, different mutations have originated from different parts of the globe. and they differ in characteristics, transmissibility and antigenicity. The changing immune profile of the human population in response to these dynamic mutations is a major factor leading to terms such as variant of concern, variant under investigation and variant of clinical significance.

The mutation factors such as pathogenicity, infectivity, transmissibility and antigenicity are prevalent in the study of virus biology. The spike protein amino acid change D614G, for example, is one of the effects of dynamic mutations. The coding sequence that occurs in the reaction demonstrates a high dN/dS ratio which is suggestive of a positive selection at codon position 614 [43,64]. Subsequent studies have indicated that D614G proffers a moderate benefit for infectivity [65,66] and transmissibility [67]. Other mutations springing up from many continents of the world have since emerged after D614G and are continually being monitored.

As these global mutations continue to unfold, vaccine scientists continue to provide the evidence signifying a reduction in the neutralization of some SARS-CoV-2 variants through postvaccination serum studies and the inquiry on how this may affect vaccine effectiveness moving forward. Moderna and other manufacturers are forging alliances on professional fronts to provide updates on vaccine sequences with the hope to combat evolving mutations. Another area of interest is the surveillance of genetic and antigenic changes in the global virus population and their impacts on phenotypic mutations.

The spike protein, being the most immunogenic part of the virus, is also responsible for the fusion of the virus to cell membranes. It is the principal target of neutralizing antibodies generated following infection by SARS-CoV-2 [68–70].

Spike receptor-binding domain (RBD) mutations and immune escape studies provide a direction for the study of how mutations in SARS-CoV-2 spike protein affect neutralization. Studies on traditional escape mutation work recognise that mutations within a virus population exposed to either mAbs [71] or convalescent plasma containing polyclonal antibodies exhibit a target characterization of a specific mutation [72–74]. A wider investigation of either large numbers of circulating variants or all possible amino acid substitutions in the RBD is yet to be concluded [75–77].

The presence of a relatively small number of RBD residues confers most mutations that reduce antibody binding effects. This signifies the presence of a substantial level of immune dominance within RBD39 [78].

In conclusion, to fully understand the impact of mutations on vaccine efficacy, the authenticity of viruses and the process of pseudoviruses possessing whether individually or in combination requires special attention. The main challenge is to understand how the larger sets of mutations and circulating spike mutations are significant when formulating postvaccination sera neutralization assays.

To develop, test and widely distribute better and updated COVID vaccines is a stimulus for a safer world during a pandemic. The article has highlighted how to create a new vaccine in future pandemics that possesses adequate immune-stimulatory effects and is also tailored to combat and be maximally cross-reactive to as many circulating emerging antigenic variants as possible.

## Discussion

In the immediate future, the approach of current vaccine manufacturers to redesign and tweak their vaccines for better protection against new mutating variants of concern would be a welcome development. This will mitigate against mutation and related efficacy issues worldwide.

On the bright side, the UK, USA, Israel and Gibraltar are experiencing positive mass vaccination effects with stable infection and hospitalisation rates. The reason for this is likely that they have been able to match the right vaccine against the strains predominant in their countries, accounting for the positive antibody response data, falling morbidity burden and stable hospitalisation rates of COVID-19 patients being experienced currently in their respective countries. This positive trend has been dampened by the resurgence of increasing worrisome mutations – the Omicron strain at present. For the reported success to continue, it is essential to put in measures that continue to prevent an influx of more vaccine-evasive strains like Omicron, Delta, South Africa, Brazilian and California strains from countries with ‘presumed’ worse disease burdens. Like South Africans, every country around the world should endeavour to conduct vaccine efficacy studies first before embarking on mass vaccination of their populace. This will enable matching of the dominant strains in respective countries to appropriate effective vaccines as the South Africans did with the single-dose Johnson & Johnson vaccine.

**Figure 6.**
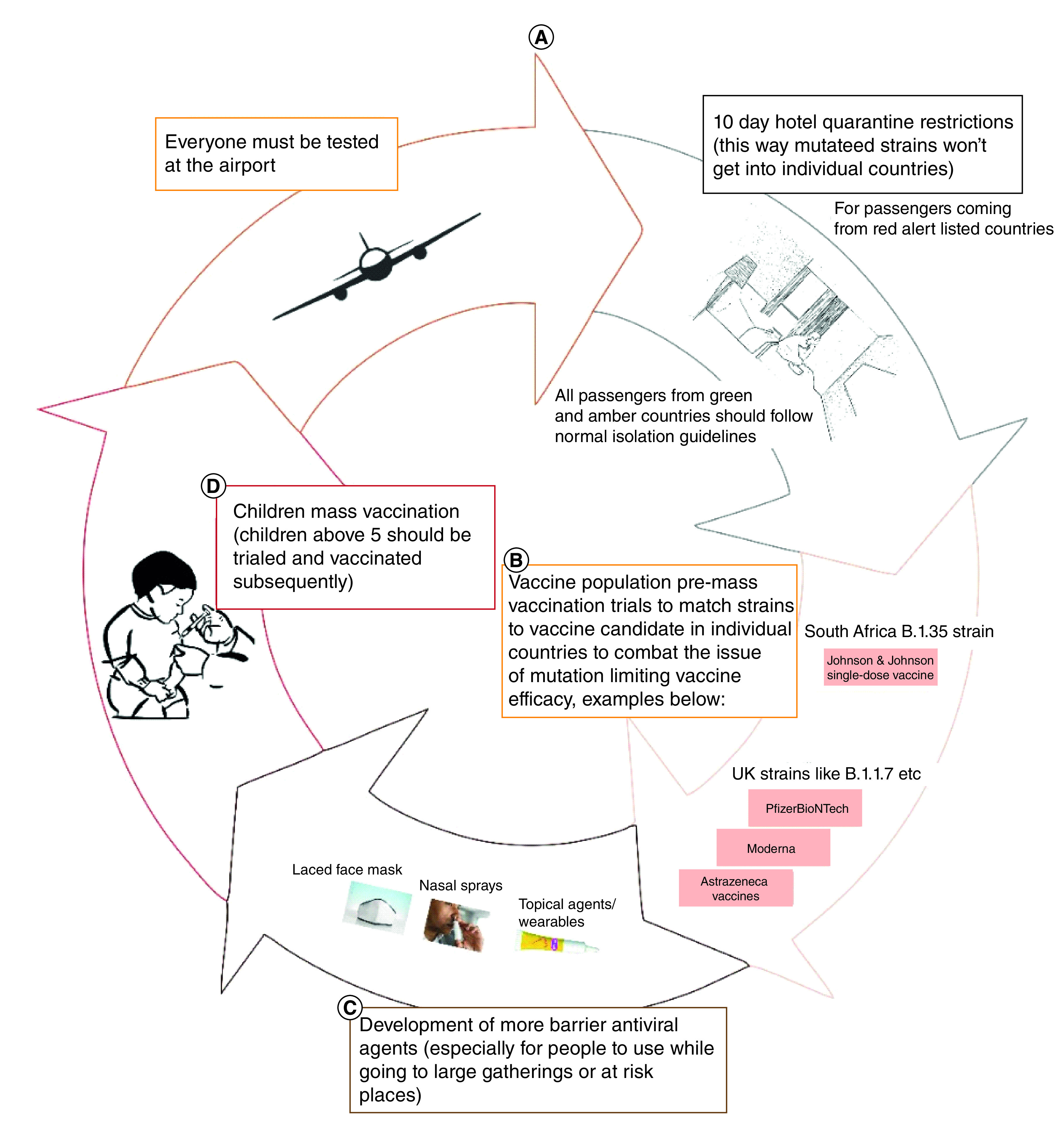
Suggested algorithm to bridle COVID-19 as mass vaccination programs continue worldwide in case mutating variants become more clinically significant.

As is being done in the UK and the rest of the western world, it is imperative to vaccinate children older than 12 years in particular, as they are potential harbingers of infection to more vulnerable adults. This is illustrated in Figure 6.

In terms of nanotechnology, more research into niosome and liposome carriers for vaccines and COVID-19-related drug development needs to be done in the coming years, as NPs are an efficient way of transporting drugs to target human cells or organs with increased efficacy and reduced toxicity. This would particularly be important if mucosal vaccines are developed against future pandemic viruses.

The UK COVID taskforce launched by the government to identify novel antiviral treatments for UK patients who have been exposed to COVID-19, in a bid to reduce the disease burden and speed up recovery time, is another welcome development. The taskforce has clinical trial platforms like AGILE, RECOVERY, REMAP-CAP, PRINCIPLE and PROTECT-CH. The latter two platforms should excite the UK Life Science industry in particular, as they afford an opportunity to develop either novel or repurposed therapeutics to prevent progression of mild to severe disease, for example, through trials of inhaled steroids like budesonide, and to treat mild to early severe COVID-19 and prevent progression to late severe or critical disease. This could also aid the development of new therapies, such as by finding appropriate agents through clinical trials to reduce viral loads in the early stages of the disease when the virus is still replicating in the nasopharynx, including gargle agents, preventive nasal barrier gels, nasal patches, nasal sprays, repurposed drugs and novel nanoemulsion therapies to reduce patients’ severe/critical drowning in mucus secretions and invariable mucus plugging. These approaches are illustrated in Figure 6.

The authors of this article, as drug-development scientists working on the development of novel COVID-related prophylactic and therapeutic agents, which possibly might be suitable for the PRINCIPLE and PROTECT-CH trial platforms based on nanotechnology techniques, would like to suggest more studies which focus on the development of barrier medical devices, including NP-driven topical agents, facemasks laced with viricidal agents, topical nasal patches and nasal sprays, among others, for use by the general populace when going to large gatherings following the removal of restrictions, as illustrated in Figure 6.

## Future perspective

Chile’s post-vaccination devastating story has shown that countries with high vaccination rates like the UK, Israel and the USA should not rest on their oars in the face of unpredictable mutations that are capable of reducing vaccine efficacy. In addition, Brazil and India have shown that in the face of poor vaccination rates and likely prevalence of potentially deadly mutant strains, even children die from COVID-19.

Therefore, the development of new national guidelines in the coming months for treating the remaining moderate-to-severe COVID-19 infections, to avoid hospitalisation or prevent progression to critical disease remains imperative. In addition, to be battle-ready, should mutations of concern become an issue in well-vaccinated populations like ours, there is a need for more research into the development of prophylactic agents against respiratory viral infections even despite the abundance of effective vaccines.

**Executive summary**

**Introduction**

- The COVID-19 pandemic was first reported in Wuhan, China, on 30 December 2019 [4] and soon became a global disease of immense significance due to global travel.
- SARS-CoV-2 is transmitted through four main routes: direct physical contact with a carrier, indirect contact, interactions with contaminated objects, and via droplet and airborne transmission, often occurring through coughs, sneezes, breathing and aerosolization, when atomised virus becomes suspended in airflow.
- The most topical issue on COVID-19 at present is that of mutant strains capable of evading the immune system and being resistant to the current vaccines.

**Current vaccine technologies**

- The inactivated vaccine approach involves using the whole SARS-CoV-2 virus, after inactivating or killing it with heat, radiation or chemicals.
- A nucleic acid vaccine delivers a specific set of instructions by encoding genes to our cells, either in the form of DNA or DNA converted to mRNA, for them to make the specific protein to trigger memory T-cell responses and the production of humoral antibodies.

**Nanoparticles & novel COVID-19 mRNA vaccine development**

- The development of mRNA-based COVID-19 vaccines was followed by many clinical trials to identify the most suitable carriers for delivering these vaccines to the host cell.
- Nanoparticles can be used as delivery systems for mRNA-based vaccines, to carry, protect and deliver the loaded mRNA molecules into the target cells after administration.

**Emerging COVID vaccine development approaches**

- As of 18 October 2021, a total of 33,942,329 vaccine doses had been administered in a country with a national population of 19.12 million.

**Discussion**

- More research into niosome and liposome carriers for vaccines and COVID-19-related drug development needs to be done in the coming years, as nanoparticles are an efficient way of transporting drugs to target human cells or organs with increased efficacy and reduced toxicity.
